# Botanicals Reduce Circulating Concentrations of Cholesterol and Triglycerides and Work Synergistically With Arachidonic Acid to Reduce Inflammatory Cytokines in Cats

**DOI:** 10.3389/fvets.2021.620447

**Published:** 2021-02-04

**Authors:** Dennis E. Jewell, Kiran S. Panickar

**Affiliations:** ^1^Department of Grain Science and Industry, Kansas State University, Manhattan, KS, United States; ^2^Hill's Pet Nutrition, Inc., Topeka, KS, United States

**Keywords:** cat, arachidonic acid, botanicals, cytokines, triglycerides, cholesterol

## Abstract

Forty Eight cats were used to measure the effects of feeding a traditional adult cat food supplemented with either arachidonic acid (ARA), a botanical mix (botanicals) or both on circulating biochemical parameters and inflammatory cytokines. The cats were healthy adults (mean age, 3.0; range, 1.3–6.4 years). The adult cats were fed one of four foods (*n* = 12 per group) for 84 days (dietary changes reported as fed): a traditional adult cat food (control, 0.05% ARA no added botanicals), or control food supplemented with arachidonic acid from chicken liver (0.13% ARA when supplemented), control food supplemented with botanicals (green tea 0.5%, fenugreek 0.05%, and tulsi 0.003%), and control plus ARA (0.13% as fed) with botanicals (green tea 0.5%, fenugreek 0.05%, and tulsi 0.003%). Response variables were compared between treatments: initially, and at 84 days (end of study). The measurements were standard complete blood counts and chemistries as well as circulating cytokines. Botanical inclusion reduced (*P* < 0.05) circulating cholesterol and triglycerides while arachidonic acid increased (*P* < 0.05) their concentrations. The pro-inflammatory cytokines MCP-1, TNFα, SDF-1, Flt3L, IL-8, IL-12p40, IL-13, and IL-18 were all reduced (*P* < 0.05) in cats after consuming the ARA + botanicals food for 84 days with little change after consuming the other foods. Therefore, this combination of ARA and botanicals may be of value in reducing inflammation.

## Introduction

The potential of manipulating feline metabolism through the supplementation of dietary polyunsaturated fatty acids (PUFA) including the feline nutritionally required arachidonic acid (ARA) is significant. This change in metabolism through dietary manipulation of PUFA levels can significantly influence health and disease (1). For example when dietary ARA was increased (with EPA and DHA) there was a change in renal function and a reduction in a renal biomarker for the risk of calcium oxalate stone formation in cats (2). Changing the concentrations of dietary PUFA in the cat may also result in a change in immune function through their effects on circulating leukotriene (LTB_5_) as well as populations and proliferations of lymphocytes. This is in relationship to dietary EPA and DHA and to a lesser degree alpha linolenic acid (3). There appears to be a reduced immune response when the dietary fatty acids EPA and DHA were increased. In the dog, cell mediated immune response was decreased as dietary EPA and DHA was increased (4). This may partially be through the response in circulating ARA which decreased, thus supplying fewer *n*-6 fatty acids and decreasing the circulating *n*-6 to *n*-3 fatty acid ratio in blood (4). This change in immune response as *n*-3 fatty acids increase as compared to an *n*-6 (specifically ARA) increase is similar to the inflammatory immune response reported in swine and poultry (5) and also the changes reported in humans (6). When a medium fat (13.2% dry matter), high *n*-3 food (7.0% of fatty acids as *n*-3 fatty acids) was compared to a high fat (27.6% dry matter), high *n*-6 (15.9% of fatty acids as *n*-6 fatty acids) food in cats there was no change in the cytokines TNFα or IL-6, or IL-10 (7). Biomarkers of stress and disease, including the influence of ARA, have recently been reviewed (8). In this review it was reported that ARA through a prostaglandin-F2a isomer (8-isoprostane) is a source of oxidation and platelet activation which would reasonably increase pro-inflammatory cytokine production. Arachidonic acid-induced pro-inflammatory cytokine production has also been shown to be dependent on prostaglandin synthase-1 activity (9). In a recent review we concluded that ARA was, in general, a pro-inflammatory fatty acid as compared to the *n*-3 fatty acids EPA and DHA (10).

Botanical extracts rich in polyphenols have antioxidant, anti-inflammatory, and anti- microbial effects (11, 12). Polyphenols are natural substances with variable phenolic structures and are enriched in vegetables, fruits, grains, bark, spices, roots, tea, and wine (13, 14). There is evidence to indicate that consumption of green tea through the botanicals it contains is beneficial in reducing blood lipids in humans although consistent beneficial effects have not always been reported (15). In a meta-analysis of human studies it was concluded that green tea intake lowered total cholesterol and low density lipoprotein cholesterol while not influencing high density lipoprotein cholesterol. However, there was a need for greater diversity within the study population and longer duration studies (16). This study in cats tests the combination of botanical extracts to exert a beneficial effect using cytokines and selected blood parameters as response criteria. In our study we chose to use a combination of green tea extract, tulsi extract (*Ocimum sanctum* or holybasil), and fenugreek extract (*Trigonella foenum-graecum*) to potentially ameliorate the pro-inflammatory effect of ARA. These extracts have been reported to have an ameliorating effect on ARA metabolism in cell culture as well as in rodent studies including green tea extract (17), tulsi extract (18, 19), and fenugreek extract (20). Therefore, this study investigated the interaction and possible synergistic effect of ARA and these three botanicals in cats. The ARA increase was chosen in an effort to double the concentration provided in the control food while the botanical mixture addition was based on previous work (unpublished) showing a benefit in reducing markers of calcium oxalate stone formation.

## Materials and Methods

### Animals

The protocols of this study were reviewed and approved by the Institutional Animal Care and Use Committee, Hill's Pet Nutrition, Inc., Topeka, KS (Study Permit Number: CP654). Individual cats were deemed available for the study if they were domestic short hair adults (over a year of age), had a normal physical examination, serum chemistry profile, complete blood count, urinalysis, total T4, and blood taurine. Cats were excluded if they had acute or chronic disease, abnormal physical examination or laboratory findings, or positive urine culture. This was established within 12 months of study initiation. Forty eight cats were assigned to one of four treatments. Treatments were defined by experimental food which was fed for 84 days. Treatment one (fed the control food) consisted of 7 neutered males and 5 spayed females with an average age of 2.8 years (range 1.3–5.4 years). Treatment two (fed the control food + botanicals) consisted of 6 neutered males and 6 spayed females with an average age of 3.1 years (range 1.3–6.5 years). Treatment three (fed the control food + ARA) consisted of 6 neutered males and 6 spayed females with an average age of 3.2 years (range 1.3–6.5 years). Treatment four (fed the control food + ARA + botanicals) consisted of 6 neutered males and 6 spayed females with an average age of 2.9 years (range 1.3–6.4 years). All cats were group housed and had access to toys, an enclosed porch with natural lighting, and water *ad libitum*. Food intake was *ad libitum* and measured by continuous collection of weight loss from electronic feeders. Each cat's intake was measured by the difference between initial and final weight after each meal. Feeders were constructed to allow only one cat to consume food at a time and multiple feeders for each food were present to allow ample access to food when desired. Food intake was recorded daily and body weight measured every week.

### Foods

Dietary treatments are shown in Table 1 with the four foods being defined by either a complete and balanced control food (Control or Group 1), control food + botanicals (Group 2), control food+ARA (Group 3), or control food + ARA + botanicals (Group 4). Nutrient composition (including fatty acids), and fiber concentrations of foods were determined by a commercial laboratory (Eurofins Scientific, Inc., Des Moines, IA). Proximate analyses were completed using the following techniques: moisture—AOAC 930.15; protein—AOAC 2001.11; fat—AOAC 954.02; fiber—AOAC 962.09; ash—AOAC 942.0. Fatty acid (FA) concentrations were determined by gas chromatography of FA methyl esters, and were expressed as g FA/100 g of food as fed. The control food consisted of rice, corn gluten meal, and poultry by-product meal with amino acids, vitamins and minerals added to make a complete and balanced food for adult cats. The ARA addition was achieved by a partial substitution of the poultry by-product meal with chicken liver to meet the ARA increase desired while the botanical mix was substituted for rice. Chicken liver was purchased from SPF/Diana Pet Food (Hodges, South Carolina, USA), green tea extract and fenugreek were purchased from Naturex (Hackensack, NJ; Numbers DA240149 and EA142400, respectively), tulsi was purchased from Sabinsa (East Windsor, NJ Number 2044).

**Table 1:** Food composition (grams/100 g as fed unless otherwise stated).

**Analyte or ingredient**	**Control**	**Control + botanicals**	**Control + ARA**	**Control + ARA + botanicals**
Liver	0	0	7.5	7.5
Green tea	0	0.5	0	0.5
Fenugreek	0	0.05	0	0.05
Tulsi	0	0.003	0	0.003
Ash	4.47	4.46	4.63	4.59
Crude Fiber	0.6	0.6	0.5	0.5
Moisture	5.96	5.77	6.11	5.94
Protein	33.17	32.75	33.88	33.81
Fat	15.68	15.75	16.47	16.37
Atwater energy*^e^* (kcal/kg)	3,898	3,908	3,830	3,932
Myristic acid (14:0)	0.15	0.15	0.15	0.14
Palmitic acid (16:0)	3.27	3.20	3.40	3.27
Palmitoleic acid (16:1)	0.45	0.44	0.45	0.44
Steric acid (18:0)	1.43	1.40	1.60	1.52
Oleic acid (18:1)	5.33	5.21	5.43	5.24
LA [18:2 (*n*-6)]	2.87	2.71	2.74	2.73
aLA [18:3 (*n*-3)]	0.13	0.12	0.12	0.12
ARA [20:4 (n-6)]	0.06	0.05	0.13	0.13
EPA [20:5 (*n*-3)]	<0.01	<0.01	<0.01	<0.01
DHA [22:6 (*n*-3)]	<0.01	<0.01	0.02	0.02

e *Energy is calculated by using modified Atwater coefficients (21)*.

### Blood Analysis

Blood samples were collected (8 mL) by jugular venous puncture under anesthesia (within 10 min of 2 mg/kg telazol IM) and serum and plasma separated by centrifugation at 30,000 g for 10 min at the beginning and end of the study on the treatment foods. Samples were analyzed immediately for biochemical profiles or stored at-−20°C until analyzed for cytokine concentrations. Serum chemistry analysis was performed on a Roche Diagnostics biochemical analyzer (Cobas 6000 series, c501 module, Indianapolis, IN). These analysis used the Roche reagent cassettes as follows: albumin—ALB2, urea—UREAL, creatinine—CREP2, glucose—GLUC3, triglycerides—TRIGL, cholesterol—CHOL2. Serum cytokine analyses were performed using the Feline Cytokine/Chemokine Magnetic Bead Panel ELISA kit (FCYTMAG-20K-PMX, Millipore-Sigma) following the manufacturer's directions. This kit measured the following 19 cytokines: sFas, TNF?, IL-12p40, SCF, PDGF-BB, IL-13, IL-18. IL-6, IL4, IL-2, GM-CSF, KC, RANTES, SDF-1, FLT-3L, IL-1β, IFN?, MCP-1, and IL-8. Briefly, 75 μl (25 μl each of serum sample, antibody-immobilized beads, and assay buffer) were incubated overnight at 4°C in a 96-well plate on a shaker. Beads were then washed twice with 200 μl wash buffer and incubated with 25 μl detection antibodies on a plate shaker for 1 h at room temperature (RT). Subsequently 25 μl streptavidin-phycoerythrin was added to each well and incubated on a shaker for another 30 min at RT. Beads were then washed twice with 200 μl wash buffer. Sheath fluid (100 μl) was added to each well and the fluorescence was detected on Luminex® 200™instrument (Luminex Corp., TX). The data were analyzed using the Milliplex Analyst software. All samples were run in duplicate.

### Statistics

Statistical analyses for serum analytes were performed using a repeated measures regression model (PROC MIXED) in SAS (Statistical Analysis Software) using the version 9.4 (SAS Institute, Cary, NC). This general linear model evaluated food effects, time, and the food by time interaction with each cat identification used as a random variable. Each cat was used as the experimental unit and used in the repeated measure analysis with initial value as a covariant. If there was a significant main effect of food by time interaction than the *post-hoc* evaluation was made to conclude what means were changing over time using a paired *t*-test. The least square means ± SEM are reported while significance was accepted as *P* < 0.05.

## Results

There was an increase in body weight in cats fed all foods except the cats fed the control and botanicals food. The cats consuming the control and botanicals food also had reduced intake when compared to the other groups (Table 2), although they consumed their daily energy requirement which was sufficient calories to not change weight. There was no change in circulating albumin or urea nitrogen concentrations (Table 2). Dietary treatment did effect circulating concentrations of glucose, creatinine, cholesterol, and triglycerides (Table 2). During the 84 days of the study there was an increased concentration of blood glucose in the control fed cats which was completely offset by the addition of botanicals, however not offset by ARA addition or the combination of ARA and botanicals. Circulating concentration of creatinine was decreased in the control fed cats while increased in the botanical supplemented fed cats and unchanged in the cats in response to ARA supplementation or the combined ARA and botanicals supplementation. There was a decreased concentration of both circulating cholesterol and triglycerides in the cats fed control and botanicals while all other cats were either unchanged or increased concentrations (Table 2).

**Table 2:** Body weight and selected serum biochemistries from serum biochemical profiles (values are lsmeans + standard errors).

**Analyte**	**Control**	**Control ± botanicals**	**Control ± ARA**	**Control ± ARA ± botanicals**
Body weight (kg) initial	5.15 ± 0.05	5.15 ± 0.05	5.15 ± 0.05	5.15 ± 0.05
Body weight (kg) 84 day	5.40 ± 0.05^*^^a^	5.09 ± 0.05^b^	5.35 ± 0.05^*^^a^	5.25 ± 0.05^a, b^
Food consumed (kcal/wt^3/4^)	77 ± 3.2^a^	59 ± 3.2^b^	72 ± 3.2^a^	68 ± 3.2^a, b^
Glucose (mg/dl) initial	89.7 ± 3.6	89.8 ± 3.6	89.8 ± 3.6	89.8 ± 3.6
Glucose (mg/dl) 84 day	101.5 ± 3.6^*^^a^	88.7 ± 3.6^b^	105.5 ± 3.5^*^^a^	97.4 ± 3.6^*^^a, b^
Albumin (mg/dl) initial	3.42 ± 0.04	3.44 ± 0.04	3.44 ± 0.04	3.42 ± 0.04
Albumin (mg/dl) 84 day	3.50 ± 0.04	3.40 ± 0.04	3.42 ± 0.04	3.44 ± 0.04
Urea nitrogen (mg/dl) initial	19.5 ± 0.6	19.9 ± 0.6	20.1 ± 0.6	19.6 ± 0.6
Urea nitrogen (mg/dl) 84 day	21.8 ± 0.6^*^	21.3 ± 0.6^*^	21.4 ± 0.6^*^	21.4 ± 0.6^*^
Creatinine (mg/dl) initial	1.19 ± 0.03	1.18 ± 0.03	1.18 ± 0.03	1.18 ± 0.03
Creatinine (mg/dl) 84 day	1.11 ± 0.03^*^^a^	1.29 ± 0.03^*^^b^	1.16 ± 0.03^a^	1.20 ± 0.03^a, b^
Triglycerides (mg/dl) initial	38.1 ± 3.8	48.6 ± 4.3	38.5 ± 3.8	37.4 ± 3.8
Triglycerides (mg/dl) 84 day	52.8 ± 3.8^*^^a^	29.4 ± 3.8^*^^b^	60.2 ± 3.8^*^^a^	54.8 ± 3.9^*^^a^
Cholesterol (mg/dl) initial	140.3 ± 4.5	140.4 ± 4.5	139.5 ± 4.5	139.5 ± 4.5
Cholesterol (mg/dl) 84 day	135.0 ± 4.5^a^	110.6 ± 4.5^*^^b^	172.6 ± 4.5^*^^c^	167.2 ± 4.5^*^^c^

* *Means were different (p ≤ 0.05) within an analyte between initial and final concentration*.

a, b, c *Means within a row with different superscripts are different*.

**Figure 1:**
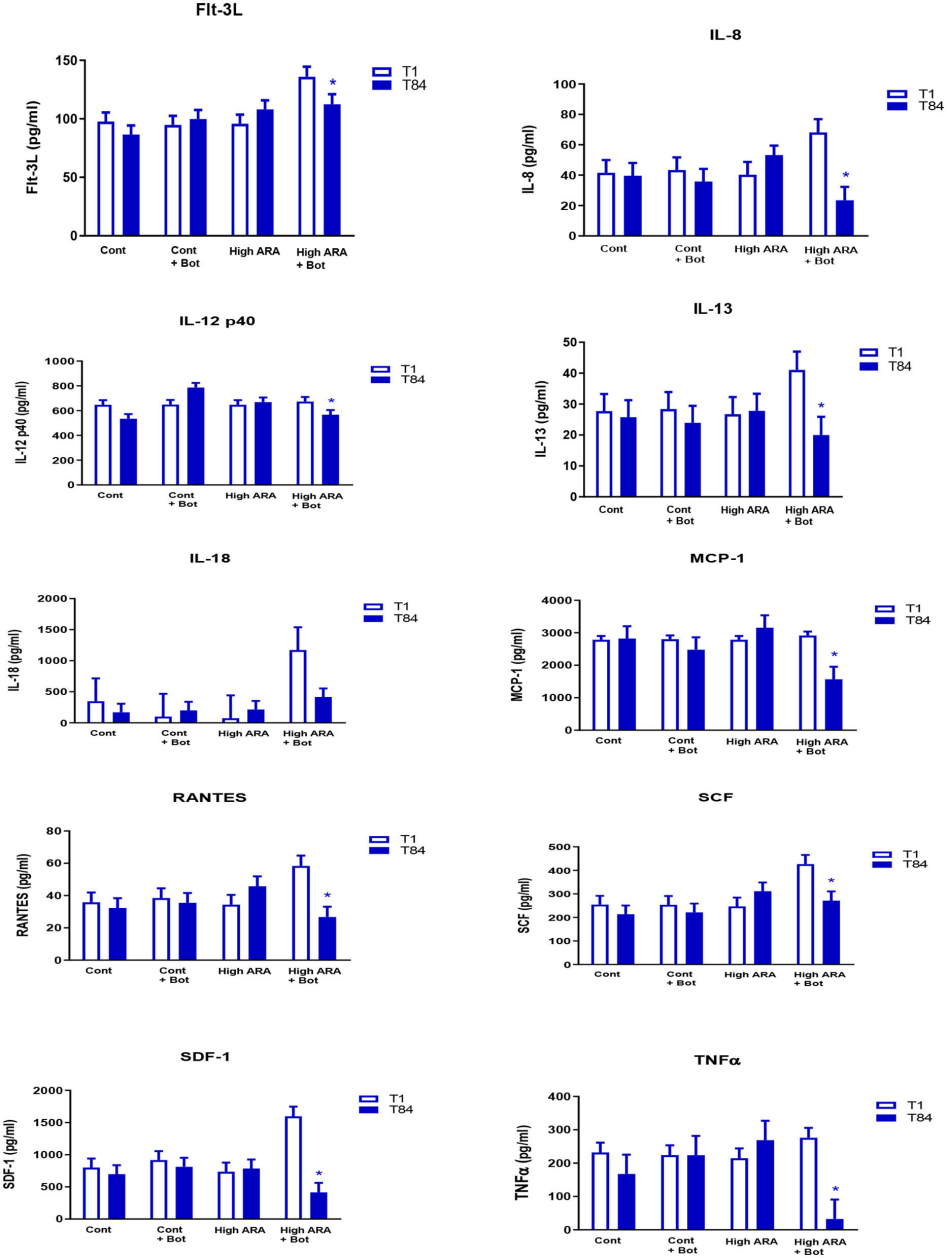
Dietary influence^e^ on circulating cytokine concentration (LSMeans with SE bars). ^e^Treatments are as follows: Cont, a traditional adult cat food (control, 0.05% ARA no added botanicals); Cont+Bot, control food supplemented with with botanicals (green tea 0.5%, fenugreek 0.05%, and tulsi 0.003%); High ARA Control food supplemented with arachidonic acid from chicken liver (0.13% ARA as fed); High ARA+Bot, control food plus ARA (0.13% as fed) with botanicals (green tea 0.5%, fenugreek 0.05%, and tulsi 0.003%). *Means are reduced from initial values. Flt-3L, Fms-like tyrosine kinase 3 ligand; IL-8, Interleukin 8; IL-12 p40, Interleukin 12 subunit p40; IL-13, Interleukin 13; IL-18, Interleukin 18; MCP-1, Monocyte Chemoattractant Protein-1; RANTES, Regulated on Activation, Normal T Expressed and Secreted; SCF, Stem Cell Factor; SDF, Stromal Derived Factor; TNFα, Tissue Necrosis Factor α.

The addition of botanicals in the presence of increased dietary ARA significantly reduced the levels of serum cytokines on day 84 when compared with day 1 while remaining unchanged in all other groups (Group 4; see Figure 1 for all cytokines). Flt-3L levels were not significantly different from day 1 to day 84 in groups 1, 2 or 3. However, in Group 4 which consumed increased ARA and botanicals, Flt-3L level was reduced significantly by 17.3% (Day 1 = 135.9 pg/ml vs. Day 84 = 112.4 pg/ml; *p* < 0.05). Similarly, for IL-8, the levels were not significantly different from day 1 to day 84 in groups 1, 2, or 3 but were reduced significantly in group 4 at day 84 (68.1 pg/ml) compared to day 1 (23.4 pg/ml; *p* < 0.05). The decline in IL-12p40 in Group 4 from day 1 (672.9 pg/ml) to day 84 (567 pg/ml) was 15.8% (*p* < 0.05). The statistically significant decline in the values of other cytokines from day 1 to day 84 are: IL-13, 51.3%; IL-18, 64.6%; MCP-1/CCL2, 46.2%; RANTES, 54.4%; SCF, 36.5%; SDF-1, 74.2%; and TNFα, 88.2%.

The cytokine GM-CSF was barely detectable in most cats in any of the groups (*n* = 2/12 in group 1; *n* = 7/12 in group 2; *n* = 1/12 in group 3; *n* = 2/12 in group 4 showed some levels of GM-CSF). We therefore did not include it in the final analysis. Similarly, there were several cats in several groups where levels of detection for IFN-γ, PDGF-BB, IL-2, IL-1β, IL-4, IL-6, and KC were very low and therefore not included in the final analysis. For instance, in Group 1, only 25% of cats (3 out of 12 cats) had IFN-γ levels that were detected at all time-points, making comparison between groups difficult and therefore excluded from the analysis. FasL was also barely detectable in control cats and importantly their levels were below detectable levels at some time point in the same cat. In short, we did not include the analytes for analysis if there were more than 40% of either the time points missing in a group (due to levels below detection).

## Discussion

The effects of ARA and botanicals on food intake and body weight were seen only in the cats consuming the botanical blend without ARA. The cats eating food with this blend (control + botanicals) had a lowered food intake (as compared to all other groups). Also, these cats did not significantly gain weight as compared to both the cats eating the control food or the food with ARA alone. This differential response was while glucose numerically declined in the cats eating the control + botanical food while all other cats had a significant increased concentration of circulating glucose. Previous work has suggested that green tea alone (22), is beneficial during weight loss in humans. It has also been reported that fenugreek supports glucose control in rats, mice, rabbits and dogs (23). This review (23) also states that there is a cholesterol lowering effect of fenugreek which may be having an effect with green tea to reduce circulating cholesterol in the cats consuming the botanical supplemented food. This current study suggests that the control of circulating glucose may also be seen in cats eating the botanical blend. The opposite effect of botanical supplementation alone on cholesterol and triglycerides was seen in the cats consuming the ARA supplemented food which resulted in increased concentrations of both cholesterol and triglycerides. The influence of dietary ARA in increasing circulating lipids has been previously reported (24) and the causal pathways have been reviewed (25) which showed a direct pathway from ARA to circulating triglycerides. The botanical effect of reducing circulating concentrations of cholesterol and triglycerides when the botanicals were supplemented in the control food was not present when botanicals were supplemented with ARA which leads to the conclusion that this botanical effect is not able to counteract the effects of ARA on circulating lipids in cats.

The effect on circulating concentrations of cholesterol and triglycerides seen with the botanicals alone was not observed in the cytokine concentration in this study. Interestingly, it was not that the botanicals simply offset the effects of ARA on circulating cytokines, rather in the presence of increased dietary ARA they are singularly effective. This suggests that there is some fatty acid pathway that potentiates the botanical response and is suggestive for further research. The anti-inflammatory effects of the botanical extracts in attenuating inflammation was expected based on their reported effects in other species (11–16). However, the robust combined effects of the three extracts with ARA on some of the cytokines was novel and not to our knowledge previously reported. This combination may be beneficial as a feline nutritional supplement. For instance, Fms-like tyrosine kinase 3 ligand (Flt-3L) is a small growth factor that increases levels of B and T cell (26) and can also increase levels of dendritic cells in mice (27). Some histiocytic proliferative disorders that occur in dogs and sometimes in cats can have the dendritic cell lineages (28). Therefore, foods that have the potential to reduce levels of FLt-3L and subsequently control dendritic cell population might be desirable in certain health conditions in cats. Some of the cytokines including IL-8, and RANTES that have been reduced in cats fed the ARA + botanical extracts have also been reported to be upregulated in cats with sepsis and septic shock (29) indicating that results from our study have the potential for influencing health conditions in cats with similar upregulation in cytokines. For example, TNFα is a cytokine that is upregulated in diabetic conditions and feline diabetes has been reported (30–32). It is possible that a diet that includes this mix of ARA and botanical extracts might be beneficial in reducing systemic inflammatory markers in diabetes in felines. Although there was no effect of the combined ARA and botanical extracts on final glucose concentration when compared to the control fed cats, there was an increased glucose concentration during the 84 days of the study in all of the cats with the exception of those fed the botanical bundle without ARA addition. Therefore, if this ARA and botanical extract was to be used to reduce systemic inflammatory markers in diabetic felines it would need to be added to a food designed to aid in the management of this disease that would also control circulating blood glucose. Parys et al. (33) reported increased serum concentrations of pro-inflammatory cytokines including CXCL12, IL-12, IL-18, and Flt3L in cats with non-obstructive acute feline idiopathic cystitis (FIC) when compared to control healthy cats, while Stella et al. (34) reported higher TNFα in FIC cats when compared to healthy controls. Long-term nutritional solutions are highly desirable in such cats to attenuate the pro-inflammatory cytokine associated with FIC. Nguyen Van et al. (35) showed that cats with intestinal inflammation had significantly more mRNA levels of IL-6, IL-10, IL-12p40, TNFα, and TGF-β than those with normal intestinal morphology. Taken together, these studies indicate a strong component of pro-inflammatory cytokines that play an important role in these adverse health conditions in cats. Diet formulations which include this combination of ARA with green tea, tulsi and fenugreek may be beneficial in improving or maintaining health in cats prone to disorders where inflammatory cytokines need to be controlled long-term.

It is interesting that the group fed botanicals alone had an increase in circulating creatinine. As creatinine is the byproduct of muscle metabolism it is influenced by a number of factors including lean body mass and glomerular filtration rate in cats (36, 37). Because these cats were not gaining weight, it is reasonable to conclude that it was not a change in body lean resulting in a change in creatinine production. Although an increased protein turnover cannot be excluded, it is most likely that there was a slight decreased glomerular filtration rate resulting in the increased circulating creatinine. This change in the group mean was not a change in the cats with highest concentration of creatinine and no individual cat was above the pre-established colony normal reference interval for creatinine. Rather, it was the result of an increased concentration, especially in cats with creatinine concentration below the normal reference interval when the study began. As the overall increase was not associated with any cat increasing above the normal colony interval, but rather influenced by cats increasing from initial concentrations below the colony reference interval, it is unlikely that the creatinine change reflected a deleterious effect of the botanical mixture.

In conclusion, the cat is responsive to both increasing dietary ARA and a mix of green tea, fenugreek and tulsi. This combination may be beneficial in reducing inflammatory cytokines while the botanical mix alone may be beneficial in weight control through control of circulating glucose, food intake and by reducing circulating cholesterol and triglycerides.

## Data Availability Statement

The original contributions generated in the study are included in the article/supplementary material, further inquiries can be directed to the corresponding author.

## Ethics Statement

The animal study was reviewed and approved by Institutional Animal Care and Use Committee, Hill's Pet Nutrition, Topeka, KS, USA (Protocol Number:CP654). Written informed consent was obtained from the owners for the participation of their animals in this study.

## Author Contributions

All authors designed and conducted the experiment, evaluated, analyzed the data, authored, edited the article, and agree to be accountable for the content of the work.

## Conflict of Interest

DJ was and KP is an employee of Hill's Pet Nutrition Inc., that sells foods to enhance health and aid in the management of disease.
